# Wnt/β-Catenin Signaling Pathway Is Strongly Implicated in Cadmium-Induced Developmental Neurotoxicity and Neuroinflammation: Clues from Zebrafish Neurobehavior and In Vivo Neuroimaging

**DOI:** 10.3390/ijms231911434

**Published:** 2022-09-28

**Authors:** Yanyi Xu, Junru Liu, Yonghui Tian, Zuo Wang, Zan Song, Kemin Li, Shengxiang Zhang, Haiyu Zhao

**Affiliations:** Gansu Key Laboratory of Biomonitoring and Bioremediation for Environmental Pollution, School of Life Sciences, Lanzhou University, No. 222 South Tianshui Road, Lanzhou 730000, China

**Keywords:** cadmium (Cd), zebrafish, neurotoxicity, neurobehavioral tests, in vivo two-photon neuroimaging, microglia, Wnt/β-catenin signaling pathway

## Abstract

Cadmium (Cd) is a toxic heavy metal and worldwide environmental pollutant which seriously threatens human health and ecosystems. It is easy to be adsorbed and deposited in organisms, exerting adverse effects on various organs including the brain. In a very recent study, making full use of a zebrafish model in both high-throughput behavioral tracking and live neuroimaging, we explored the potential developmental neurotoxicity of Cd^2+^ at environmentally relevant levels and identified multiple connections between Cd^2+^ exposure and neurodevelopmental disorders as well as microglia-mediated neuroinflammation, whereas the underlying neurotoxic mechanisms remained unclear. The canonical Wnt/β-catenin signaling pathway plays crucial roles in many biological processes including neurodevelopment, cell survival, and cell cycle regulation, as well as microglial activation, thereby potentially presenting one of the key targets of Cd^2+^ neurotoxicity. Therefore, in this follow-up study, we investigated the implication of the Wnt/β-catenin signaling pathway in Cd^2+^-induced developmental disorders and neuroinflammation and revealed that environmental Cd^2+^ exposure significantly affected the expression of key factors in the zebrafish Wnt/β-catenin signaling pathway. In addition, pharmacological intervention of this pathway via TWS119, which can increase the protein level of β-catenin and act as a classical activator of the Wnt signaling pathway, could significantly repress the Cd^2+^-induced cell cycle arrest and apoptosis, thereby attenuating the inhibitory effects of Cd^2+^ on the early development, behavior, and activity, as well as neurodevelopment of zebrafish larvae to a certain degree. Furthermore, activation and proliferation of microglia, as well as the altered expression profiles of genes associated with neuroimmune homeostasis triggered by Cd^2+^ exposure could also be significantly alleviated by the activation of the Wnt/β-catenin signaling pathway. Thus, this study provided novel insights into the cellular and molecular mechanisms of Cd^2+^ toxicity on the vertebrate central nervous system (CNS), which might be helpful in developing pharmacotherapies to mitigate the neurological disorders resulting from exposure to Cd^2+^ and many other environmental heavy metals.

## 1. Introduction

Cadmium (Cd), a highly toxic heavy metal and worldwide environmental pollutant, is being released into the ecosystems with the acceleration of industrialization [1]. Many countries have very strict standards for Cd in water. The European Groundwater Directive required the EU member states to set threshold values for Cd in groundwater, and these values were determined ranging from 0.08 to 27 μg/L. The WHO Guidelines for Drinking-Water Quality recommended a guideline value for Cd of 3 μg/L [2]. China [3], the European Union [4], and the United States set the maximum contaminant level for Cd to 5 μg/L [5]. In addition, the environmental quality standard for Cd in groundwater is 10 μg/L in China and Japan, and 0.5 μg/L in Denmark [4,6]. Although the content of Cd in water is strictly monitored all over the world, its concentrations still exceed the standard in many areas. The latest surveys revealed that the concentrations of Cd^2+^ range from 0.1 to 0.3 μg/L in various water systems and can even reach 300 μg/L in polluted rivers [7,8,9,10]. Specifically, the concentration of Cd in groundwater around waste disposal sites in the United States is as high as 6000 μg/L and leachates from municipal solid waste landfills in the European Union can reach up to 2700 μg/L [11,12]. This heavy metal which is able to enter bodies from the digestive system and respiratory system [13] has been reported to have a half-life of about 15 to 20 years in humans [14,15]. Due to its widespread existence and extremely long biological half-life, extensive studies have been focused on its toxicity to humans and various organisms. In particular, considerable evidence has shown that Cd exposure and accumulation increase the risks of neurological disorders. For example, Cd^2+^ has been demonstrated to impair nervous tissue and different cell types including neurons and glial cells [16,17,18], thus representing one of the risk factors of neurodegenerative diseases including Parkinson’s disease and Alzheimer’s disease [14,19,20]. In addition, studies based on different animal models revealed significant associations between nerve damage and environmental exposure to Cd^2+^ [21,22,23,24]. To gain more insights into the mechanisms of the pathogenesis of nerve damage caused by Cd, numerous molecular, biochemical, and structural studies have been carried out, and fundamental progress has been achieved. For instance, Cd^2+^ are likely to bind to various proteins and regulatory elements, thereby disrupting redox homeostasis and communication of signals [25]. The Cd^2+^-induced increase of oxidative stress can be considered an etiological factor in a variety of nervous system diseases [26,27,28]. In brief, Cd has been commonly recognized to pose great threats to the central nervous system (CNS). Nevertheless, the cellular and molecular basis of the Cd-induced developmental neurotoxicity remains largely unknown.

Among the various molecular components and pathways involved in neurodevelopment, the Wnt/β-catenin signaling pathway is essential and has been extensively investigated [29,30]. Briefly, the core component of the classical Wnt signaling pathway is β-catenin [31]. When this pathway is activated, the activated β-catenin accumulate in the cytoplasm and are further transferred to the nucleus, where β-catenin bind to the transcription factor Tcf/Lef to regulate the expression of Wnt target genes [32,33]. As an evolutionarily conserved molecular pathway, the Wnt/β-catenin signaling pathway plays important roles in embryonic development and many physiological processes [34]. For instance, it is crucial in the growth, differentiation, apoptosis, and self-renewal of cells [35,36]. Previous studies have demonstrated that this pathway is involved in neurogenesis and vascular repair, and it is often aberrantly activated during tumorigenesis [37,38,39]. In addition, some toxicant-induced developmental defects have been demonstrated to be achieved through the disruption of the Wnt signaling pathway [40,41]. Importantly, activation of the Wnt signaling pathway has been reported to inhibit pathological neuroinflammation [42]. Thus, in combination with previous studies and our recent findings, we hypothesized that the developmental neurotoxicity induced by Cd^2+^ exposure could be closely related to the Wnt/β-catenin signaling pathway. However, knowledge of this crucial pathway and its role in the Cd^2+^-caused neurotoxicity is still very limited.

Zebrafish, as a classical vertebrate model system with a well-established genetic and developmental background, possesses significant advantages including strong fertility, transparent embryos, small size, rapid development, convenient drug delivery, and importantly, sufficient homology with humans at the genetic level. Furthermore, various well-constructed transgenic lines expressing fluorescent proteins offer powerful tools for behavioral tracking of diverse cell types [43,44]. Therefore, it has been widely applied in recent toxicological studies [45,46]. One of our recent studies showed that early development of zebrafish was delayed, and neurobehavioral patterns including spontaneous activity and reactivity to different environmental signals were significantly disturbed during developmental Cd^2+^ exposure. Importantly, we revealed associations between Cd toxicity and neurodevelopmental disorders as well as microglia-mediated neuroinflammation [47]. However, the underlying neurotoxic mechanisms remain largely unclear. In this follow-up study, taking advantage of the zebrafish model in high-throughput behavioral tests, in vivo CNS imaging, as well as genetic and pharmacological manipulation, we explored the role of the Wnt/β-catenin pathway in the process of Cd^2+^-induced neurodevelopmental and neurological disorders. The results of this study may not only provide novel insights into the cellular/molecular mechanisms of Cd^2+^ toxicity on vertebrate CNS, but also be very helpful to exploiting pharmacotherapies to mitigate the neurological disorders resulting from exposure to environmental Cd, as well as many other environmental heavy metals.

## 2. Results

### 2.1. Wnt/β-Catenin Signaling Is Declined upon Cd^2+^ Exposure in Zebrafish Larvae

**Figure 1:**
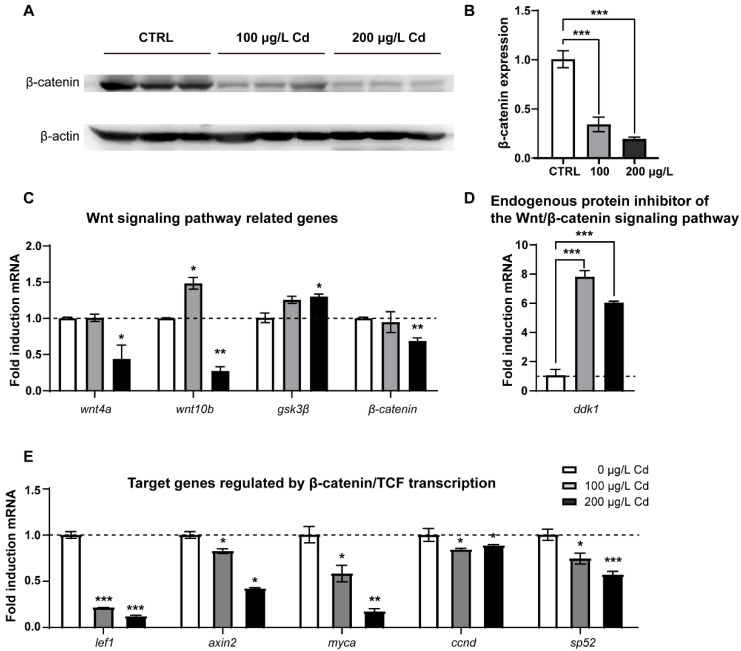
Cd^2+^ exposure inhibited zebrafish Wnt/β-catenin signaling pathway. (**A**,**B**) Western blot analysis and quantitation of β-catenin expression levels in 6 dpf zebrafish larvae exposed to different levels of Cd^2+^. (**C**–**E**) qRT-PCR analysis of relative expression of genes involved in Wnt/β-catenin signaling pathway. The values are presented as the mean ± SEM. The results of either nonparametric test or ANOVA followed by multiple comparison test are listed in Table S2. “*” indicates significant differences (* *p* < 0.05, ** *p* < 0.01, *** *p* < 0.001).

To assess the effects of Cd^2+^ exposure on the Wnt/-catenin signaling pathway, we examined the expression levels of Wnt-related proteins and genes in 6 dpf zebrafish larvae exposed to different levels of Cd^2+^. Six days after Cd^2+^ exposure, the content of β-catenin significantly decreased with the increase in Cd^2+^ levels (Figure 1A,B). To further understand the molecular basis of the Cd^2+^-modulated Wnt signaling pathway in zebrafish, the relative expression of key genes (*wnt4a*, *wnt10b*, *gsk3β*, and *β-catenin*), endogenous inhibitor *ddk1*, and target genes (*lef1*, *axin2*, *myca*, *ccnd*, *sp52*) were determined by qRT-PCR analyses. The results showed that Cd^2+^ exposure significantly downregulated the expression of *wnt4a*, *wnt10b*, *β-catenin*, *lef1*, *axin2*, *myca*, *ccnd*, and *sp52*, while it upregulated the expression of *ddk1* and *gsk3β* (Figure 1C–E). Thus, the results of the protein and gene expression analyses consistently revealed that Cd^2+^ exposure could, to some extent, repress Wnt/β-catenin signaling in zebrafish larvae.

### 2.2. Activation of Wnt Signaling Pathway can Attenuate the Damaging Effects of Cd^2+^ on Zebrafish Early Development

**Figure 2:**
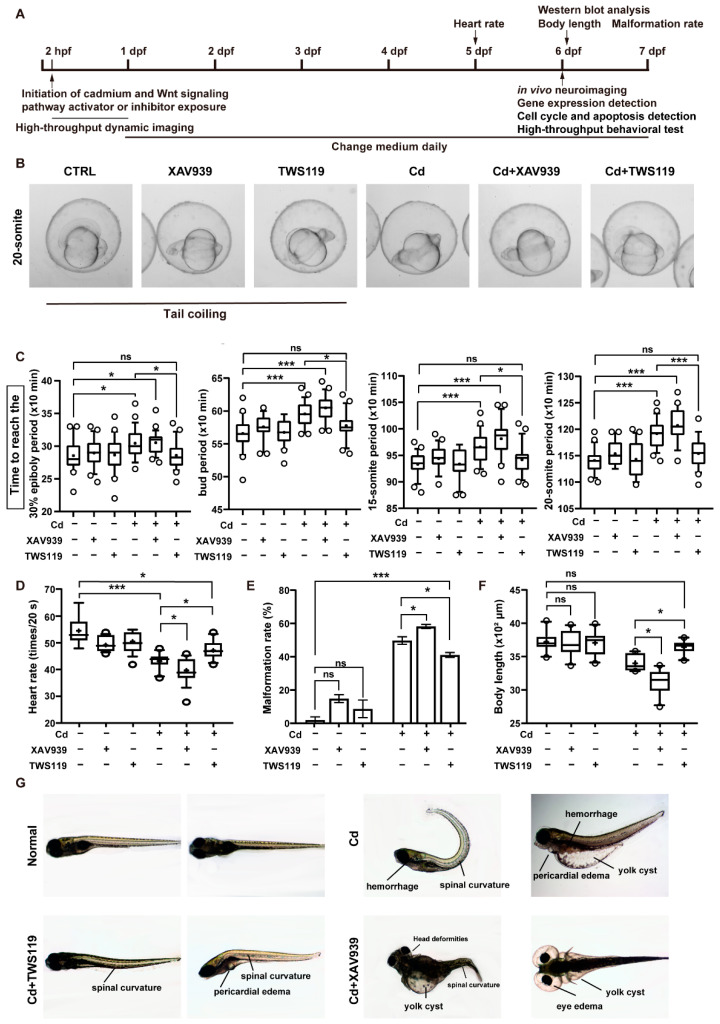
Activation of Wnt signaling pathway attenuated the adverse effects of Cd^2+^ on zebrafish early development. (**A**) Schematic design for the following study. (**B**,**C**) High-throughput tracking (representative images) of zebrafish early development, and the duration for embryos in different groups to reach certain stages. (**D**–**F**) Heart rate, rate of deformed individuals, and body length of 6 dpf zebrafish in different groups. (**G**) Representative morphological characteristics of zebrafish larvae in each group. The values are presented in boxplots (median and mean, whiskers show 90% confidence levels) or in histograms (mean ± SEM). The results of either nonparametric test or ANOVA followed by multiple comparison test are listed in Table S2. “*” indicates significant differences (* *p* < 0.05, *** *p* < 0.001).

Next, in order to elucidate the importance of Wnt/β-catenin signaling in Cd^2+^-induced developmental toxicity, we added an additional pharmacological activator (TWS119) and an inhibitor (XAV939) of the Wnt signaling pathway to the culture medium of zebrafish larvae coexposed to 200 μg/L of Cd^2+^ and continuously analyzed their early developmental indicators at different stages up to 7 dpf (Figure 2A). Importantly, the expression of key factors involved in the Wnt/β-catenin signaling pathway at both gene and protein levels demonstrated the effectiveness of the activator and inhibitor in the zebrafish model (Figure S1A–C). Consistent with our previous study, Cd^2+^-exposed zebrafish embryos showed significant developmental delay at different stages, whereas activation of Wnt/β-catenin signaling significantly attenuated this adverse effect, and inhibition of this pathway, to some extent, aggravated this developmental retardation (Figure 2B,C and Figure S2). The dynamic early development of zebrafish embryos in different groups is shown in Video S1. Particularly, both the normal control group and the Cd + TWS119 group reached the stage of spontaneous coiling earlier than the Cd^2+^-exposed and Cd + XAV939 groups. Similarly, activation of the Wnt signaling pathway significantly dampened the adverse effects of Cd^2+^ on zebrafish heartbeat, as well as normal growth and development (Figure 2D–F). The morphological characteristics of the zebrafish larvae in different groups are present in Figure 2G.

### 2.3. Activation of Wnt Signaling Pathway Alleviates the Inhibitory Effects of Cd^2+^ on Zebrafish Activity and Reactivity

**Figure 3:**
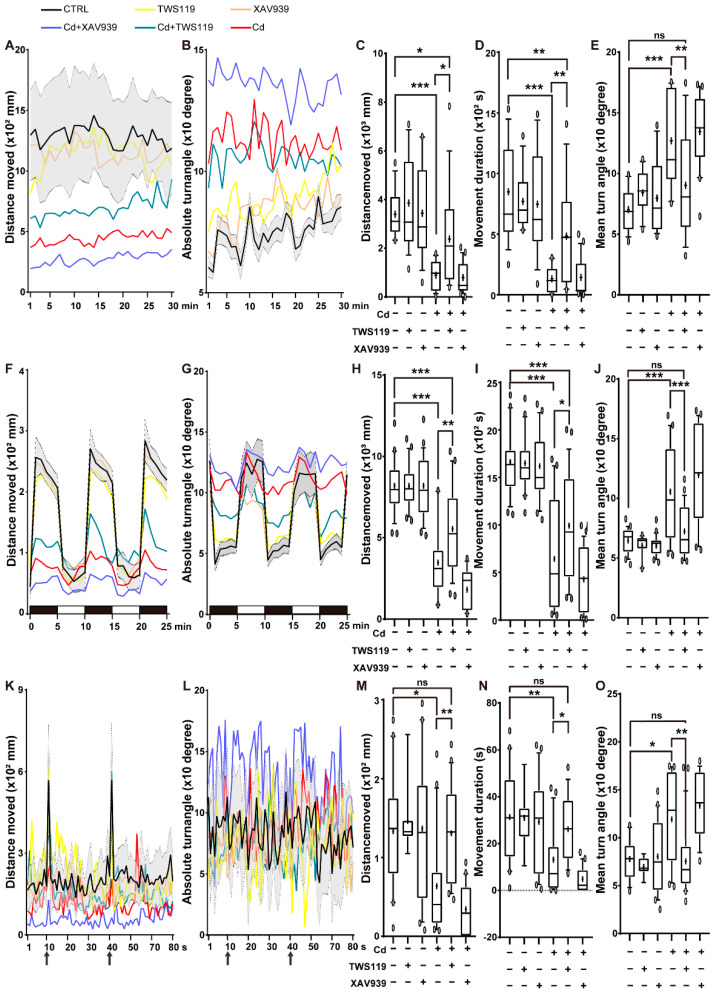
Patterns of zebrafish locomotor activities and reactivity to light–dark/vibration stimulation. Swimming distance, velocity of 6 dpf zebrafish larvae in open field tests (**A**–**E**), subjected to light–dark cycles (**F**–**J**) and vibration stimulation (as indicated by the arrows) (**K**–**O**). Panels (**A**,**B**,**F**,**G**,**K**,**L**) were plotted in 5 min/10 s time interval, panels (**C**–**E**,**H**–**J**,**M**–**O**) were plotted with average values of the whole-tracking. Each test was performed at least three times. The numerical values are presented in line charts or boxplots (median and mean, whiskers show 90% confidence levels). The results of either nonparametric test or ANOVA followed by multiple comparison test are listed in Table S2. “*” indicates significant differences (* *p* < 0.05, ** *p* < 0.01, *** *p* < 0.001).

The behavior and activity, as well as reactivity to various environmental signals, reflect the neurodevelopmental state of the zebrafish model [48]. Therefore, we recorded the locomotor activity and response to light–dark/vibration stimulation of 6 dpf zebrafish larvae in different groups to illustrate the involvement of the Wnt/β-catenin signaling pathway in the Cd^2+^-induced neurodevelopmental injury. In all the tests including the locomotion tests (Figure 3A–E and Figure S3A–C), light–dark stimulation tests (Figure 3F–J and Figure S3D–F), and vibration stimulation tests (Figure 3K–O and Figure S3G–I), the distance moved, duration of movement, and maximum acceleration of the Cd^2+^-exposed zebrafish larvae were all significantly reduced. Interestingly, upon activation of the Wnt/β-catenin signaling pathway via coexposure with TWS119, their vitality increased significantly. In addition, the remarkable reduction in movement duration as well as the augmentation in turn angle of Cd^2+^-exposed individuals indicated that these zebrafish larvae, instead of swimming evenly, were more apt to twitch irregularly. However, this inducing effect of abnormal neurobehavior was significantly alleviated by cotreatment with TWS119, the classical Wnt activator. Thus, our results suggest that the Cd-induced repression of the Wnt/β-catenin signaling pathway results in the neurobehavioral disorders of zebrafish larvae.

### 2.4. Activation of Wnt Signaling Pathway Alleviates Cd^2+^-Induced Neurodevelopmental Disorders

**Figure 4:**
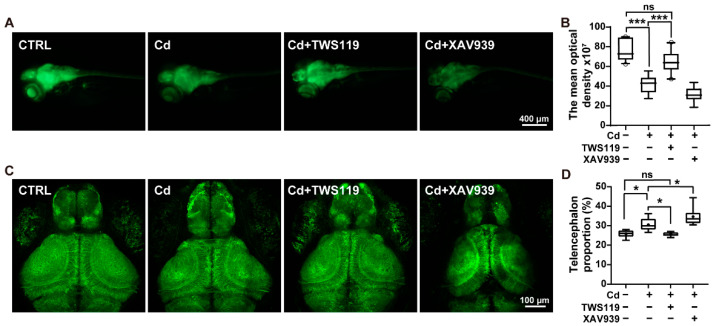

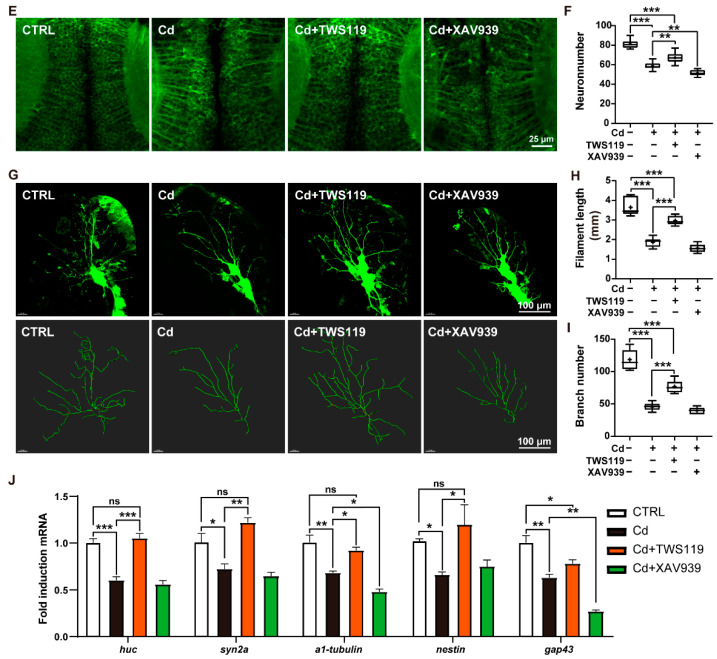
Involvement of zebrafish Wnt signaling pathway in Cd^2+^-induced neurodevelopmental disorders. (**A,B**) Fluorescence imaging of 6 dpf *Tg(elavl3:EGFP)* larvae and statistics of relative fluorescence intensity. (**C**,**D**) Confocal fluorescence imaging of the telencephalon and midbrain regions, as well as the statistics of telencephalon proportion. (**E**,**F**) Imaging of the PVL region in 6 dpf *Tg(elavl3: EGFP)* larvae and density statistics of PVN. (**G**–**I**) Imaging of trigeminal ganglion neurons and structural analysis in 24 hpf zebrafish embryos. (**J**) Relative expression of genes related to neurodevelopment and differentiation. In histograms, values are represented as mean ± SEM, while in boxplots, values are represented as median and mean, and whiskers show 90% confidence levels. The results of nonparametric test and ANOVA, as well as multiple comparison test are listed in Table S2. “*” indicates significant differences (* *p* < 0.05, ** *p* < 0.01, *** *p* < 0.001).

In order to explore the neural structure basis of the above neurobehavioral detection results, the neuron specifically labelled *Tg(elavl3:EGFP)* zebrafish line was applied and imaging was performed initially under the standard fluorescence microscope. The results showed that the fluorescence intensity of the brain and spine region in the 6 dpf larvae decayed in the Cd^2+^-exposed individuals, whereas this effect was significantly attenuated in the Cd^2+^ and TWS119 coexposed larvae (Figure 4A,B). Next, a finer and clearer structure of neurons was then obtained by using the in vivo two-photon neuroimaging. In accordance with our previous data, the area of telencephalon tended to be expanded in the Cd^2+^-exposed, as well as the Cd^2+^+XAV939 co-exposed groups, while this teratogenic effect on the brain was significantly alleviated upon TWS119 cotreatment (Figure 4C,D). Moreover, in our recent study, we identified a significant decrease in the density of periventricular neurons in the tectal PVL (periventricular layer) region of the zebrafish larvae during developmental Cd^2+^ exposure. However, this distinct tendency towards neuron loss was significantly reversed by TWS119 cotreatment, and aggravated by XAV939 coexposure (Figure 4E,F). Next, for the purpose of further verifying the involvement of the Wnt signaling pathway in the Cd^2+^-affected zebrafish early neurogenesis, trigeminal ganglion neurons in 24 hpf embryos were also examined in detail via in vivo two-photon imaging. Interestingly, the structural complexity of the TG neurons was reduced upon Cd^2+^ exposure, whereas this effect was significantly restored in the Cd^2+^+TWS119 cotreatment embryos, as indicated by our analysis of filament length and branch number (Figure 4G–I). To explore the molecular/genetic basis of the above imaging data, the expression profiles of genes including *elavl3* (*huc*), *nestin*, *α1-tubulin, syn2a*, and *gap43*, which are closely involved in neuron development and differentiation, were detected by qRT-PCR analysis. As expected, the expression of these genes was significantly downregulated in Cd^2+^-exposed 6 dpf larvae, while this trend was remarkably reversed upon activation of the Wnt signaling pathway by TWS119 (Figure 4J). Thus, these results demonstrate that the Cd^2+^-modulated Wnt/β-catenin pathway contributes to the observed neurodevelopmental disorders.

### 2.5. Activation of Wnt Signaling Pathway Restores Cd^2+^-Induced Neuronal Apoptosis and Cell Cycle Arrest

**Figure 5:**
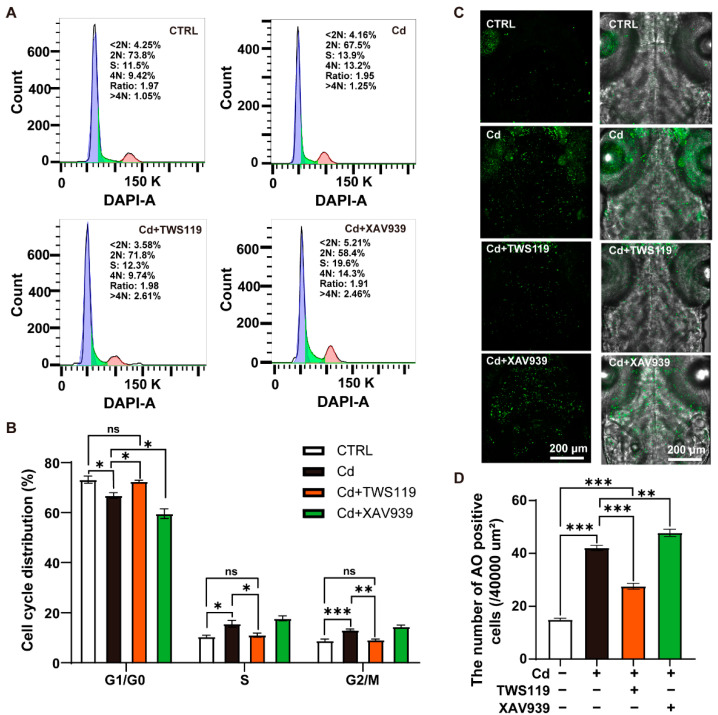

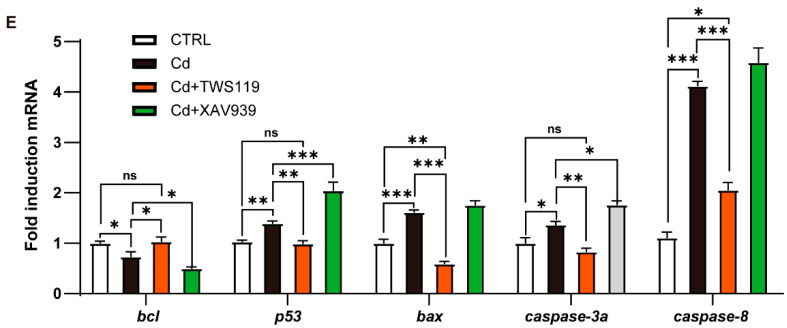
Apoptosis and cell cycle arrest in zebrafish larvae caused by Cd^2+^ are mediated by Wnt signaling pathway. (**A**,**B**) Flow cytometry analysis and distribution of cell cycle phase of neurons and neuronal precursor cells. (**C**,**D**) Vertical view in the brain of AO-stained zebrafish larvae (6 dpf) and statistics of apoptotic cell numbers. (**E**) Relative expression analysis of genes related to cell apoptosis. In histograms, values are represented as mean ± SEM, while in boxplots, values are represented as median and mean, and whiskers signify 90% confidence interval. The results of nonparametric test and ANOVA, as well as multiple comparison test are listed in Table S2. “*” indicates significant differences (* *p* < 0.05, ** *p* < 0.01, *** *p* < 0.001).

It is well-known that the Wnt signaling pathway plays significant roles in regulating cell cycle. To further explore the cellular basis of the Cd^2+^-induced neurodevelopmental deficits, as well as the involvement of the Wnt signaling pathway, neurons and neuronal precursor cells specifically labeled by GFP in 6 dpf *Tg(elavl3:EGFP)* zebrafish larvae were selected, and their cell cycle distributions were determined by using flow cytometry. Our results showed that Cd^2+^ exposure significantly reduced the number of cells in the G1/G0 phase, increased the proportion of cells in the G2/M phase, and remarkably blocked cells in the S phase, which was highly consistent with our previous results, indicating that Cd^2+^ exposure leads to neuron-related cell cycle arrest. However, upon activation of the Wnt signaling pathway via TWS119 coexposure, the proportion of cells in the G1/G0 phase increased to a similar level as the normal control group, while the blockage of cells in the G2/M phase and the S phase was to some extent released (Figure 5A,B). Further, acridine orange (AO) staining was performed to examine apoptosis in the brains of zebrafish larvae in different groups. Consistently, a few scattered apoptotic cells were observed in the brain region of larvae in the normal control group, while a significant induction of apoptosis was detected in the brain region of the Cd^2+^-exposed larvae. Interestingly, this apoptosis-inducing effect was significantly reversed by TWS119 cotreatment, and aggravated by XAV939 coexposure (Figure 5C,D). In addition, the results of qRT-PCR analysis showed that, compared with the normal control group, the expression of proapoptotic genes including *p53*, *bax*, *caspase-3a*, and caspase-8 was significantly induced while the antiapoptotic gene *bcl2* was repressed by Cd^2+^ exposure. The activator of the Wnt signaling pathway TWS119 significantly attenuated these alternations while the inhibitor XAV939 to some extent exacerbated these trends (Figure 5E). Therefore, our results suggest that the inhibition of the Wnt signaling pathway is one of the key mechanisms of Cd^2+^-induced neuronal apoptosis and cell cycle arrest.

### 2.6. Activation of Wnt Signaling Pathway Inhibites Cd^2+^-Induced Microgliosis and Neuroinflammation

**Figure 6:**
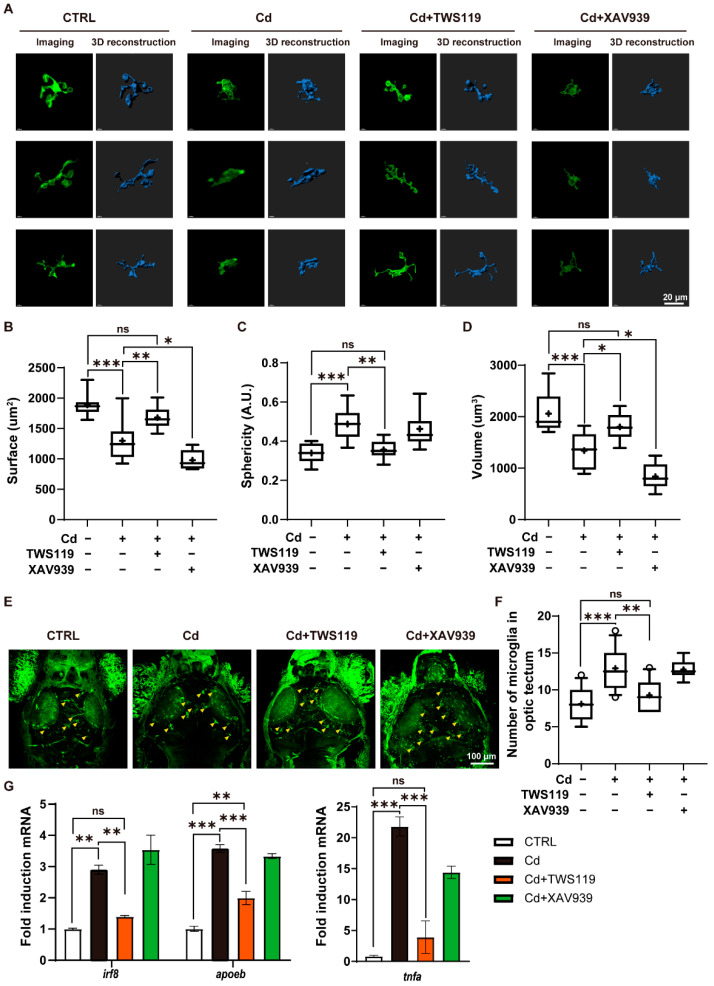
Role of zebrafish Wnt signaling pathway in Cd^2+^-induced microgliosis and neuroinflammation. (**A**–**D**) Morphological alterations of microglia in 6 dpf *Tg(ApoE-EGFP)* zebrafish larvae, and quantification of morphological parameters. (**E**,**F**) In vivo neuroimaging and microglia counting in the optic tectum region of 6 dpf zebrafish larvae. (**G**) Expression patterns of genes closely related with microgliosis and neuroinflammatory response. The numerical values are presented as either mean ± SEM in histograms, or median and mean, while whiskers show 90% confidence levels in boxplots. The results of either nonparametric test or ANOVA followed by multiple comparison test are listed in Table S2. “*” indicates significant differences (* *p* < 0.05, ** *p* < 0.01, *** *p* < 0.001).

Our previous study demonstrated that Cd^2+^ induces microglia activation and neuroinflammation in the brains of zebrafish larvae. Given that the Wnt signaling pathway has been reported to significantly regulate neuroinflammatory responses [49], we, thus, investigated the role of this pathway in Cd^2+^-induced microgliosis and neuroinflammation. Through in vivo two-photon imaging of the microglia-specific *Tg(ApoE: EGFP)* transgenic line, we observed and verified that upon Cd^2+^ exposure, the microglia in the brain of zebrafish larvae showed distinct branch retraction, changing their morphology from ramified to amoeboid, which is a typical morphological change of microglia activation and neuroimmune response. Compared with the Cd^2+^ exposed larvae, the microglia in the Cd + TWS119 group tended to possess longer branches and were more like those in the normal control group, while the microglia in the Cd + XAV939 group remained amoeboid (Figure 6A). According to the quantitative statistics of their morphologic parameters, we found that in comparison to the Cd^2+^-exposed group, the surface area and volume of microglia were significantly increased in the Cd + TWS119 group, and reduced in the Cd + XAV939 group, while the statistics for sphericity showed the exact opposite trend, indicating that activation of the Wnt signaling pathway can inhibit the activation of microglia (Figure 6B–D). In addition, during the 6-day exposure to Cd^2+^, an induction of microglia density was also observed in the midbrain optic tectum of the zebrafish larvae. However, this Cd^2+^-induced microgliosis was significantly repressed upon TWS119 coexposure (Figure 6E,F). Finally, the expression patterns of genes related to microglia development and inflammatory responses were determined by qRT-PCR analysis. *Irf8*, a crucial gene for microglia development, and *apoeb*, a typical microglia marker gene, as well as the gene encoding proinflammatory factor tnf-α, were all significantly upregulated by Cd^2+^ exposure. However, these inductive effects were significantly attenuated by the activation of the Wnt signaling pathway via TWS119 treatment (Figure 6G). Together, our results suggest that the Wnt signaling pathway is strongly implicated in microglia polarization and proliferation, as well as their mediated neuroinflammatory response.

## 3. Discussion

Cadmium (Cd), as a toxic and ubiquitous heavy metal pollutant, is easily accumulated in organisms, and brings great risks to human health and ecosystems. In recent decades, Cd has been widely recognized as one of the neurotoxic agents associated with many neurological diseases. It may alter the release of neurotransmitters and lead to changes in the blood–brain barrier (BBB) by inducing oxidative stress [28]. It may alter calcium homeostasis, lead to DNA damage [50] and lipid peroxidation [51,52,53]. However, the mechanisms of Cd^2+^-induced neurotoxicity have not been fully investigated.

Zebrafish is a famous model system in the study of genetics and developmental biology [54,55]. Because of its conserved relationship with human beings in CNS structure and function, zebrafish is currently one of the most valuable model systems used to study neurotoxicity induced by various environmental pollutants [56]. Recently, taking full advantages of zebrafish in live neuroimaging and neurobehavioral tracking, we performed in-depth studies of the effects of environmental levels of Cd^2+^ on the early development, structure, and function of the CNS [47], and on this basis, we explored the molecular mechanisms of Cd^2+^-induced neurotoxicity. Briefly, in this study, we focused on the implications of the Wnt/β-catenin signaling pathway, and revealed that (1) Cd^2+^ exposure significantly affected the expression of key factors in the zebrafish Wnt/β-catenin signaling pathway; (2) pharmacological activation of the Wnt/β-catenin signaling pathway significantly repressed the Cd^2+^-induced cell cycle arrest and apoptosis, thereby attenuating the inhibitory effects of Cd^2+^ on early development, behavior, and activity, as well as the neurodevelopment of zebrafish larvae; (3) activation and proliferation of microglia, as well as the altered expression profiles of genes associated with neuroimmune homeostasis triggered by Cd^2+^ exposure, could also be significantly alleviated by the activation of the Wnt/β-catenin signaling pathway.

Numerous articles reported that the Wnt/β-catenin signaling pathway plays crucial roles in many biological processes including neurodevelopment, cell survival, and cell cycle regulation, as well as microglial activation [57,58,59]. For instance, previous works demonstrated that the canonical Wnt/β-catenin pathway plays key roles in embryonic development and drives the angiogenesis process in the CNS, inducing the vascular system and specialized BBB [60,61,62]. The differentiation of embryonic stem cells into different germ layers requires the activation of Wnt/β-catenin signaling to steer cells towards the mesendoderm lineage or inactivation to obtain the neuroectoderm [63]. The study of Gary Davidson et al. demonstrated that Wnt components are strongly involved in the regulation of mitotic events, indicating that cell cycle and Wnt signaling are closely related [64]. Juliette Van Steenwinckel et al. revealed that the Wnt signaling pathway regulates microglial activation, which is critical in the neuroinflammatory response caused by brain injury and diseases [42]. Waldo Cerpa et al. showed that this pathway also functions in synaptic transmission and activity-dependent synaptic plasticity [65]. In addition, our previous studies showed that Cd^2+^ impairs the early neurodevelopment of zebrafish by inducing cell cycle arrest and apoptosis [47]; therefore, naturally, we speculated that the damaging effects of Cd^2+^ to the CNS development of zebrafish might be closely related to the Wnt/β-catenin signaling pathway.

Interestingly, in our study, the relatively high concentrations of Cd^2+^ inhibited the Wnt/β-catenin signaling pathway, which is contrary to the traditional view that Cd activates the Wnt/β-catenin signaling pathway and induces cancer [34,66,67]. We, thus, hypothesized that long-term exposure to low concentrations of Cd may activate the Wnt/β-catenin signaling pathway, whereas higher concentrations of Cd inhibit this pathway. This hypothesis is consistent with the results of other studies. For instance, on one hand, Prabir K Chakraborty et al. found that chronic Cd^2+^-exposure-induced renal fibrosis and/or cancer might be mediated by the activation of the Wnt signaling pathway [68,69]. Yifan Zhao et al. found that low doses of Cd activated the canonical Wnt signaling pathways and impaired hematopoietic stem cell function in mice [70]. On the other hand, Latifa Knani et al. revealed that high doses of Cd disrupted bone metabolism by inhibiting Wnt/β-catenin signaling [71]. Therefore, in this study, we further explored the role of the Wnt/β-catenin signaling pathway in Cd^2+^-induced neurotoxicity by adding an activator (TWS119) and an inhibitor (XAV939). Our results showed that the early developmental impairments induced by Cd^2+^ exposure in zebrafish embryos/larvae were significantly reduced when Wnt activators were added. Neuroimaging of the transgenic zebrafish *Tg(elavl3: EGFP)* line showed that activation of the Wnt/β-catenin signaling pathway could also reverse the nerve damage caused by Cd^2+^ exposure. Consistently, Miryam A. Fragoso et al. reported that the Wnt signaling pathway protects retinal ganglion cells from damage caused by high pressure, oxidative stress, and hypoxia [72]. The role of the Wnt/β-catenin signaling pathway in microglia has been proposed in the model of various CNS diseases in recent years [73,74,75]. Researchers demonstrated that activation of the Wnt/β-catenin signaling pathway can reduce the inflammatory responses of microglia and improve learning/memory, thereby reducing Alzheimer’s disease [42]. Therefore, we explored the role of Wnt signaling pathways on the Cd^2+^- induced microglia activation by using the *Tg(ApoE:EGFP)* line. The results showed that activation of Wnt signaling pathways could indeed significantly reduce the degree of microglia activation and neuroinflammation in the brain. Thus, our results indicated that the Wnt/β-catenin signaling pathway is strongly implicated in the developmental neurotoxicity and neuroinflammation induced by Cd exposure, but whether the Wnt signaling pathway is a direct target of Cd still needs further investigations.

## 4. Materials and Methods

### 4.1. Reagents and Chemicals

Cadmium chloride (CdCl_2_) was purchased from Sigma-Aldrich. The 1 mM stock solution was prepared in deionized water and stored at −20 °C. The working solution was obtained by diluting the stock solution in E3 embryo medium (0.15 M NaCl, 5 mM KCl, 0.25 mM Na_2_HPO_4_, 0.45 mM KH_2_PO_4_, 1.3 mM CaCl_2_, 1.0 mM MgSO_4_, and 4 mM NaHCO_3_ [pH 7.2]). To modulate Wnt signaling activities, zebrafish embryos/larvae were treated with the inhibitor XAV939 (S1180, Selleck Chem, Houston, TX, USA) and activator TWS119 (T126075, Aladdin, Shanghai, China). Tricaine (MS-222, E10521) and 1-pheny1–2-thio-urea (PTU, P7629) were purchased from Sigma-Aldrich (Bayville, NJ, USA).

### 4.2. Ethics Statement and Zebrafish Maintenance

Zebrafish husbandry and experimental procedures were reviewed and approved by the Ethics Committee of the School of Life Sciences, Lanzhou University (Approval No. EAF2020007). A great deal of effort was made to minimize animal suffering. The AB (wild-type) line and transgenic *Tg(elavl3:EGFP)* line [76] were obtained from the China Zebrafish Resource Center (CZRC). The *Tg(ApoE:EGFP)* line [77] was gifted from Prof. Jiulin Du group (Institute of Neuroscience, CAS). Zebrafish were maintained in water circulating systems (Thmorgan, Beijing, China) at 28 ± 0.5 °C with a photoperiod of 14 h light/10 h dark cycle and were fed twice a day with freshly hatched brine shrimps, as reported previously [78,79].

### 4.3. Cd^2+^ Treatment and Developmental Toxicity Assays on Zebrafish Model

Newly-hatched zebrafish embryos were collected and washed with standard E3 embryo medium within one-hour postfertilization, and then were examined under the stereomicroscope (Olympus, Japan). Normally developing embryos were exposed to CdCl_2_, or in combination with Wnt agonist (3 μM TWS119)/inhibitor (3 μM XAV939) suspended in E3 medium, which was refreshed daily. The morphological characteristics of zebrafish embryos/larvae were recorded under the stereomicroscope at different developmental stages ranging from 2 hpf to 144 hpf. Briefly, mortality as well as other parameters including hatchability, body length, cardiac rate, and malformation of embryos/larvae were recorded as previously reported [47]. In addition, the early developmental process (from 2.5 hpf to 24 hpf) of zebrafish embryos was continuously video recorded and then analyzed through the high-throughput dynamic imaging system (DynaPlant, YPHbio, Beijing, China) in the Experiment Center of the School of Life Sciences, according to the user’s manual [80].

### 4.4. Neurobehavioral Tests in Zebrafish Larvae

To start, 6 dpf zebrafish larvae without any observable morphological abnormalities were randomly selected from each group for neurobehavioral tests, as previously described [47,81]. Briefly, they were placed individually in each well of the 48 or 96 well-plates, and then were allowed to adapt in the observation chamber of the DanioVision zebrafish Tracking System (Noldus IT, Wageningen, The Netherlands) for about 15 min. Their locomotor activities, as well as swimming behaviors in response to light–dark (5:5 min L:D) and vibration stimuli (every 30 s) were recorded at 27.8 °C. Each test was repeated at least three times, and behavioral parameters including swimming distance, velocity, cumulative duration, and acceleration were all analyzed via the EthoVision XT 15.0 software (Noldus IT, Wageningen, The Netherlands). The data were presented as a moving average of larval fish in each group, during the whole test, or in 1 s/1 min time bins.

### 4.5. Western Blot (WB) Analysis

The 6 hpf zebrafish larvae (×100 per sample) were collected and homogenized in RIPA Buffer (R0278, Sigma-Aldrich, NJ, USA) containing protease inhibitors (Cat No.30827-99-7, Roche, Basel, Switzerland). Samples were centrifuged at 10,000× *g*, 4 °C for 10 min, and stored at −80 °C. An equal amount of protein (25 μg) determined via BCA assay (TaKaRa, Osaka City, Osaka Prefecture, Japan) was diluted with Laemmli buffer (Bio-Rad) containing 2-mercaptoethanol (Sigma-Aldrich). The Western blotting was performed by using a standard protocol [82]. In short, 25 g of protein were subjected to the SDS-PAGE and transferred on a PVDF membrane (Roche) by semidry blotting. After blocking with 5% BSA in TBST, membranes were incubated overnight with rabbit anti-β-catenin (1:1000; Cell Signaling) or rabbit anti-β-actin (1:2000; Cell Signaling) antibodies at 4 °C, followed by an additional hybridization for 1 h with HRP-conjugated goat antirabbit antibodies (1:2000; Cell Signaling). For visualization, membranes were exposed by using a chemiluminescence meter.

### 4.6. In Vivo Imaging of the Central Nervous System of Zebrafish Larvae

The *Tg(elavl3:EGFP)* and *Tg(ApoE:EGFP)* zebrafish lines were used for in vivo imaging of neurons and microglia, respectively, located in the brain region of zebrafish larvae. Briefly, larvae without any morphological abnormalities were washed with E3 buffer, anesthetized by using 0.01% MS-222 (Sigma-Aldrich), and then were immobilized in 1% low melting-point agarose, with their head region exposed. Imaging of neurons in the *Tg(elavl3:EGFP)* larvae were initially conducted by using a standard epifluorescence microscope (Olympus BX51). Furthermore, the two-photon confocal laser scanning microscope (Olympus FV1000) was used to image the fine structure and morphology of neurons and microglia in the telencephalon and optic tectum region. All the imaging data were processed by using the ImageJ software (v1.8.0.172, NIH) and were presented in single stack/z-stacks. Three-dimensional reconstruction and morphological analyses of neurons and microglia were performed by using the IMARIS software (V9.0.1, Bitplane, Zurich, Switzerland) based on the user guide.

### 4.7. Acridine Orange (AO) Staining and Apoptosis Analysis

Cell apoptosis in the brain of zebrafish larvae was detected by staining of acridine orange (AO) [83]. Briefly, zebrafish larvae (6 dpf) in different groups were washed twice with PBS, and then were transferred into AO (10 Μm, Sigma-Aldrich) dissolved in PBS in constant darkness for 30 min. Next, the larvae were washed twice again with PBS, anesthetized in 0.01% MS-222, and then were immobilized in 1% low melting-point agarose, with their head region exposed. Fluorescence imaging was conducted by using the confocal laser scanning microscope (Olympus FV1000) at 488 nm. Images were processed and analyzed via the ImageJ software (NIH).

### 4.8. Flow Cytometry: Analysis of Cell Cycle Distribution

Analyses of the cell cycle distribution of neurons and neuronal precursor cells were performed as previously reported [47,84]. Briefly, *Tg(elavl3:EGFP)* zebrafish larvae (6 dpf, 100 larvae per sample) which had been digested and homogenized, were washed twice with ice-cold PBS, and then were fixed in 70% ethanol at 4 °C for overnight. Next, fixed cells were washed with PBS at 4 °C for two times, resuspended in RNase A solution, and then were stained with DAPI (C0065, Solarbio, Beijing, Chian). Subsequently, EGFP specifically labeled neurons and neuronal precursors were sorted, and the distribution of cell cycles were determined via the FITC channel by using the FACSCalibur flow cytometer (BD Biosciences, Franklin Lakes, NJ, USA). Finally, the data were processed and analyzed by using the FlowJo Version 7.6.1 software in accordance with the manufacturer’s instructions.

### 4.9. qRT-PCR: Analysis of Relative Gene Expression

Total RNA was isolated from zebrafish larvae (50 larvae per sample) with the TRIzol reagent (15596018, Invitrogen, Shanghai, China) following the manufacturer’s instructions, and cDNA synthesis was performed by using the reverse transcriptional kit (CW0741, CoWin Biosciences, Jiangsu, China). Quantitative real-time PCR (qRT-PCR) was performed with SYBRGreen master mix kit (CW0659, CoWin Biosciences, Jiangsu, China), using the StepOnePlus™ (Applied Biosystems, Waltham, MA, USA) Real-Time PCR system in the Experiment Center of the School of Life Sciences according to the manufacturers’ recommendations. The primer sequences of tested genes are listed in Table S1. We calculated the relative expression levels of each gene using the 2^−ΔΔCT^ method and normalized them according to the relative expression of *β-actin*.

### 4.10. Statistical Analyses

GraphPad Prism 8.0 Software was used to perform statistical analyses in this study. Numerical results are presented in histograms as mean plus standard errors and in boxplots as median and mean, with whiskers showing 90% confidence levels. All data were initially checked for homoscedasticity and normality. In order to determine significant differences, the nonparametric test or analysis of variance (ANOVA) followed by multiple comparison tests were conducted. All the detailed statistical information has been listed in Table S2.

## Data Availability

The data presented in this study are available on request from the corresponding author.

## Supplementary Materials

The supporting information can be downloaded at: https://www.mdpi.com/article/10.3390/ijms231911434/s1.
