# Supplementary material for: Wnt/β-Catenin Signaling Pathway Is Strongly Implicated in Cadmium-Induced Developmental Neurotoxicity and Neuroinflammation: Clues from Zebrafish Neurobehavior and In Vivo Neuroimaging
Source: Int J Mol Sci. 2022 Sep 28;23(19):11434. doi: 10.3390/ijms231911434 (PMC9570071; doi:10.3390/ijms231911434)
Supplement: Supplementary file 1 [file ijms-23-11434-s001.zip › Supplementary material 2022-9-22.pdf]

Supplementary Material

Wnt/β-Catenin Signaling Pathway Is Strongly Implicated in Cadmium-induced Developmental Neurotoxicity and Neuroinflammation: Clues from Zebrafish Neurobehavior and In Vivo Neuroimaging

Yanyi Xu †, Junru Liu †, Yonghui Tian, Zuo Wang, Zan Song, Kemin Li,

Shengxiang Zhang and Haiyu Zhao*

School of Life Sciences, Gansu Key Laboratory of Biomonitoring and Bioremediation for Environmental Pollution, Lanzhou University, No. 222 South Tianshui Road, Lanzhou 730000, China.

* Correspondence: zhaohy@lzu.edu.cn; Tel.: +86 18153646061

† These authors contributed equally to this work

Table S1. Primers used for qRT-PCR analysis.

	Gene name	Sequences of the primers (5' – 3')
**Reference gene**	*β-actin*	F: CACTGAGGCTCCCCTGAATCCC
		R: CGTACAGAGAGAGCACAGCCTGG
**Neurodevelopment related genes**	*α1-tubulin*	F: AATCACCAATGCTTGCTTCGAGCC
		R: TTCACGTCTTTGGGTACCACGTCA
	syn2a	F: GTGACCATGCCAGCATTTC
		R: TGGTTCTCCACTTTCACCTT
	elavl3	F: AGACAAGATCACAGGCCAGAGCTT
		R: TGGTCTGCAGTTTGAGACCGTTGA
	nestin	F: CTTCAACATCTTCAGGCCCAAG
		R: GTGTTGGTCTGTCGATTCTCAG
	gap43	F: GCAGCAGGAAGTGGAGAAGCCA
		R: GGATTCCTCAGCAGCGTCTGGT
**Apoptosis related genes**	*bcl*	F: AACCCAAATTCTGCGCAACG
		R: ATCTACCTGGGACGCCATCT
	p53	F: GGGCAATCAGCGAGCAAA
		R: ACTGACCTTCCTGAGTCTCCA
	bax	F: GGCTATTTCAACCAGGGTTCC
		R: TGCGAATCACCAATGCTGT
	caspase-3a	F: GTTCTTTATTCAGGCTTGTCGAG
		R: TTTCTCCAGGAGTAATAACCTGG
	caspase-8	F: TGTCTCAGCCTATAGAAAGATGC
		R: AAATGAAGTAGATCTTCCCAGCT
	irf8	F: CAAAAGCCCAGATTTTGAGG
		R: TCTTTTACGGTGGTGACTGT
	apoeb	F: GCAGATGACGTGAAGAACCG
		R: GTTGCTACGGTGTTGCGGAT
	tnf-a	F: TCACGCTCCATAAGACCCAG
		R: GATGTGCAAAGACACCTGGC
**Wnt signaling pathway related genes**	*Wnt4a*	CAATGCGAGCAACTGGCTATAC
		AATGCAGCTTCCCTCGTACCTT
	β-catenin	GGAGCTCACCAGCTCTCTGT
		TAGCTTGGGTCGTCCTGTCT
	gsk3β	TCTGCTCACCGTTTCCTTTC
		CTCCGACCCACTTAACTCCA
	Wnt10b	TCCTGAAACAGGCTCGAAGT
		GCTGCTCACTTGCACACA
	ddk1	ATGCCAGAGACACTAAATGAACA
		TATGAAGGAAACCAGTTGAAAAA

	lef1	AATGATCCCGTTCAAAGACG
		CGCTAAGTCTCCCTCCTCCT
	axin2	AGCACTGGAGAAAGGCTACAGGTC
		GGAGTCATCCGTCAGTGCATC
	ccnd	CGTGTCAGGAACAGATCGAG
		TCAAATGTTAATGTCTCTGACGT
	myca	AGAAAGCTGGAGTCCTCGAC
		CTGCTGCAGTGTGTTCAGC
	Sp51	GGAGGTCACGTTGAGGATGG
		GCGACAGCGACGAGTAGAGC

Table S2. Detailed statistical information in this study.

Experiment (Figure)	Test method		Significance		N
β-catenin expression (Figure. 1B)		One-way ANOVA	***	F=42.14 P<0.001	
	CTRL	Dunnett's multiple comparisons			3
	100 ug/L vs. CTRL	-	***	<0.001	
	200 ug/L vs. CTRL	-	***	<0.001	
Wnt signaling pathway related genes (Figure. 1C)	***wnt4a***	One-way ANOVA	*	F=8.252 P=0.019	3
	100 ug/L vs. CTRL	Dunnett's multiple comparisons	ns	0.998	
	200 ug/L vs. CTRL	-	*	0.023	
	wnt10b	One-way ANOVA	**	F=108.4 P=0.002	
	100 ug/L vs. CTRL	Dunnett's multiple comparisons	*	0.017	
	200 ug/L vs. CTRL	-	**	0.005	
	gsk3β	One-way ANOVA	*	F=5.913 P=0.031	
	100 ug/L vs. CTRL	Dunnett's multiple comparisons	ns	0.052	
	200 ug/L vs. CTRL	-	*	0.046	
	β-catenin	One-way ANOVA	**	F=8.848 P=0.005	
	100 ug/L vs. CTRL	Dunnett's multiple comparisons	ns	0.811	
	200 ug/L vs. CTRL	-	**	0.004	
Endogenous protein inhibitor of the Wnt/ß-catenin signaling pathway (Figure. 1D)	***ddk1***	One-way ANOVA	***	F=101.3 P<0.001	3
	100 ug/L vs. CTRL	Dunnett's multiple comparisons	***	<0.001	
	200 ug/L vs. CTRL	-	***	<0.001	

**Target genes regulated by ß-catenin/TCF transcription (Figure. 1E)**	***lef1***	One-way ANOVA	***	F=368.9 P<0.001	
	100 ug/L vs. CTRL	Dunnett's multiple comparisons	***	<0.001	
	200 ug/L vs. CTRL	-	***	<0.001	
	axin2	One-way ANOVA	***	F=40.38 P<0.001	
	100 ug/L vs. CTRL	Dunnett's multiple comparisons	*	0.042	
	200 ug/L vs. CTRL	-	***	<0.001	
	myca	One-way ANOVA	**	F=21.62 P=0.002	
	100 ug/L vs. CTRL	Dunnett's multiple comparisons	*	0.022	
	200 ug/L vs. CTRL	-	**	0.001	
	ccnd	One-way ANOVA	*	F=7.579 P=0.023	
	100 ug/L vs. CTRL	Dunnett's multiple comparisons	*	0.015	
	200 ug/L vs. CTRL	-	*	0.042	
	sp52	One-way ANOVA	***	F=18.80 P<0.001	
	100 ug/L vs. CTRL	Dunnett's multiple comparisons	*	0.018	
	200 ug/L vs. CTRL	-	***	<0.001	
**Time to reach different period (Figure. 2C)**	**30%-epiboly**	One-way ANOVA	**	F=3.934 P=0.002	
	Cd				25
	CTRL vs. Cd	Dunnett's multiple comparisons	*	0.023	22
	XAV939 vs. Cd	-	ns	0.116	21
	TWS119 vs. Cd	-	*	0.034	22
	Cd+XAV939 vs. Cd	-	ns	>0.999	23

	Cd+TWS119 vs. Cd	-	*	0.028	22
	**bud**	One-way ANOVA	***	F=11.85 P<0.001	
	Cd				23
	CTRL vs. Cd	Dunnett’s multiple comparisons	***	<0.001	21
	XAV939 vs. Cd	-	**	0.01	21
	TWS119 vs. Cd	-	***	<0.001	22
	Cd+XAV939 vs. Cd	-	ns	0.476	21
	Cd+TWS119 vs. Cd	-	*	0.025	22
	**15-somite**	One-way ANOVA	***	F=10.65 P<0.001	
	Cd				23
	CTRL vs. Cd	Dunnett’s multiple comparisons	***	<0.001	22
	XAV939 vs. Cd	-	*	0.046	21
	TWS119 vs. Cd	-	***	<0.001	22
	Cd+XAV939 vs. Cd	-	ns	0.223	22
	Cd+TWS119 vs. Cd	-	*	0.022	22
	**20-somite**	One-way ANOVA	***	F=20.45 P<0.001	
	Cd				24
	CTRL vs. Cd	Dunnett’s multiple comparisons	***	<0.001	22
	XAV939 vs. Cd	-	***	<0.001	21

	TWS119 vs. Cd	-	***	<0.001	22
	Cd+XAV939 vs. Cd	-	ns	0.244	22
	Cd+TWS119 vs. Cd	-	***	<0.001	22
**Heart rate at 5 dpf (Figure. 2D)**		One-way ANOVA	***	F=26.14 P<0.001	15
	Cd				
	CTRL vs. Cd	Dunnett's multiple comparisons	***	<0.001	
	XAV939 vs. Cd	-	***	<0.001	
	TWS119 vs. Cd	-	***	<0.001	
	Cd+XAV939 vs. Cd	-	*	0.022	
	Cd+TWS119 vs. Cd	-	*	0.015	
**Embryo malformation rate at 7 dpf (Figure. 2E)**		One-way ANOVA	***	F=37.54 P<0.001	10
	Cd				
	CTRL vs. Cd	Dunnett's multiple comparisons	***	<0.001	
	XAV939 vs. Cd	-	***	<0.001	
	TWS119 vs. Cd	-	***	<0.001	
	Cd+XAV939 vs. Cd	-	*	0.034	
	Cd+TWS119 vs. Cd	-	*	0.023	
**Body length at 6 dpf (Figure. 2F)**		One-way ANOVA	***	F=28.99 P<0.001	
	Cd				10
	CTRL vs. Cd	Dunnett's multiple comparisons	***	<0.001	10
	XAV939 vs. Cd	-	***	<0.001	10

	TWS119 vs. Cd	-	***	<0.001	14
	Cd+XAV939 vs. Cd	-	*	0.015	10
	Cd+TWS119 vs. Cd	-	*	0.023	11
**Distance moved of spontaneous activity of larvae (Figure. 3C)**		One-way ANOVA	***	F=14.35 P<0.001	
	Cd				18
	CTRL vs. Cd	Dunnett’s multiple comparisons	***	<0.001	15
	XAV939 vs. Cd	-	***	<0.001	18
	TWS119 vs. Cd	-	***	<0.001	18
	Cd+XAV939 vs. Cd	-	ns	>0.999	21
	Cd+TWS119 vs. Cd	-	*	0.015	18
**Movement time of spontaneous activity of larvae (Figure. 3D)**		One-way ANOVA	***	F=17.42 P<0.001	
	Cd				18
	CTRL vs. Cd	Dunnett’s multiple comparisons	***	<0.001	15
	XAV939 vs. Cd	-	***	<0.001	17
	TWS119 vs. Cd	-	***	<0.001	12
	Cd+XAV939 vs. Cd	-	ns	>0.999	21
	Cd+TWS119 vs. Cd	-	**	0.007	17
**Mean turn angle of spontaneous activity of larvae (Figure. 3E)**		One-way ANOVA	***	F=12.40 P<0.001	
	Cd				17
	CTRL vs. Cd	Dunnett’s multiple comparisons	***	<0.001	15
	XAV939 vs. Cd	-	***	<0.001	18

	TWS119 vs. Cd	-	**	0.004	11
	Cd+XAV939 vs. Cd	-	ns	0.922	21
	Cd+TWS119 vs. Cd	-	**	0.005	17
**Distance moved of larvae under light-dark stimulation (Figure. 3H)**		One-way ANOVA	***	F=39.43 P<0.001	
	Cd				18
	CTRL vs. Cd	Dunnett's multiple comparisons	***	<0.001	24
	XAV939 vs. Cd	-	***	<0.001	24
	TWS119 vs. Cd	-	***	<0.001	21
	Cd+XAV939 vs. Cd	-	ns	0.067	17
	Cd+TWS119 vs. Cd	-	**	0.003	24
**Movement time of larvae under light-dark stimulation (Figure. 3I)**		One-way ANOVA	***	F=38.91 P<0.001	
	Cd				24
	CTRL vs. Cd	Dunnett's multiple comparisons	***	<0.001	24
	XAV939 vs. Cd	-	***	<0.001	24
	TWS119 vs. Cd	-	***	<0.001	21
	Cd+XAV939 vs. Cd	-	ns	0.297	24
	Cd+TWS119 vs. Cd	-	*	0.025	24
**Mean turn angle under light-dark stimulation (Figure. 3J)**		One-way ANOVA	***	F=23.20 P<0.001	
	Cd				24
	CTRL vs. Cd	Dunnett's multiple comparisons	***	<0.001	24
	XAV939 vs. Cd	-	***	<0.001	24

	TWS119 vs. Cd	-	***	<0.001	18
	Cd+XAV939 vs. Cd	-	ns	0.208	24
	Cd+TWS119 vs. Cd	-	***	<0.001	24
**Distance moved under vibration stimulation (Figure 3M)**		Kruskal-Wallis	***	H=41.33 P<0.001	
	Cd				20
	CTRL vs. Cd	Dunn's multiple comparisons	*	0.01	20
	TWS119 vs. Cd	-	*	0.024	8
	XAV939 vs. Cd	-	*	0.011	24
	Cd+TWS119 vs. Cd	-	**	0.009	21
	Cd+XAV939 vs. Cd	-	ns	0.81	22
**Movement duration under vibration stimulation (Figure 3N)**		Kruskal-Wallis	***	H=41.53 P<0.001	
	Cd				23
	CTRL vs. Cd	Dunn's multiple comparisons	**	0.005	21
	TWS119 vs. Cd	-	*	0.041	8
	XAV939 vs. Cd	-	*	0.019	23
	Cd+TWS119 vs. Cd	-	*	0.035	19
	Cd+XAV939 vs. Cd	-	ns	0.485	24
**Mean turn angle under vibration stimulation (Figure 3O)**		Kruskal-Wallis	***	H=32.6 P<0.001	
	Cd				21
	CTRL vs. Cd	Dunn's multiple comparisons	*	0.045	18
	TWS119 vs. Cd	-	*	0.039	8

	XAV939 vs. Cd	-	*	0.019	24
	Cd+TWS119 vs. Cd	-	**	0.003	22
	Cd+XAV939 vs. Cd	-	ns	>.999	17
**The mean optical density at 6 dpf (Figure. 4B)**		One-way ANOVA	***	F=41.13 P<0.001	
	Cd				8
	CTRL vs. Cd	Dunnett’s multiple comparisons	***	<0.001	11
	Cd+TWS119 vs. Cd	-	***	<0.001	10
	Cd+XAV939 vs. Cd	-	ns	0.079	9
**Telencephalon proportion (Figure. 4D)**		One-way ANOVA	***	F=13.51 P<0.001	
	Cd				
	CTRL vs. Cd	Dunnett’s multiple comparisons	*	0.026	
	Cd+TWS119 vs. Cd	-	*	0.019	7
	Cd+XAV939 vs. Cd	-	*	0.048	
**Neuron number/mm^2 (Figure. 4F)**		One-way ANOVA	***	F=69.66 P<0.001	
	Cd				
	CTRL vs. Cd	Dunnett’s multiple comparisons	***	<0.001	
	Cd+TWS119 vs. Cd	-	**	0.001	8
	Cd+XAV939 vs. Cd	-	**	0.008	
**Filament length (Figure. 4H)**		One-way ANOVA	F=4.995 P=0.012	*	
	Cd				5

	CTRL vs. Cd	Dunnett's multiple comparisons	0.957	ns	
	Cd+TWS119 vs. Cd	-	0.233	ns	
	Cd+XAV939 vs. Cd	-	0.022	*	
**Branch number (Figure. 4I)**		One-way ANOVA	F=4.509 P=0.018	*	
	Cd	Dunnett's multiple comparisons			5
	CTRL vs. Cd		0.95	ns	
	Cd+TWS119 vs. Cd	-	0.212	ns	
	Cd+XAV939 vs. Cd	-	0.036	*	
**Neurodevelopment related genes (Figure. 4J)**	***a1-tublin***	One-way ANOVA	***	F=27.18 P<0.001	3
	CTRL vs. Cd	Dunnett's multiple comparisons	**	0.003	
	Cd+TWS119 vs. Cd	-	*	0.015	
	Cd+XAV939 vs. Cd	-	*	0.036	
	syn2a	One-way ANOVA	***	F=17.32 P<0.001	
	CTRL vs. Cd	Dunnett's multiple comparisons	*	0.031	
	Cd+TWS119 vs. Cd	-	**	0.001	
	Cd+XAV939 vs. Cd	-	ns	0.748	
	elavl3	One-way ANOVA	***	F=35.02 P<0.001	
	CTRL vs. Cd	Dunnett's multiple comparisons	***	<0.001	
	Cd+TWS119 vs. Cd	-	***	<0.001	
	Cd+XAV939 vs. Cd	-	ns	0.818	

	nestin	One-way ANOVA	*	F=5.954 P=0.013	
	CTRL vs. Cd	Dunnett's multiple comparisons	*	0.049	
	Cd+TWS119 vs. Cd	-	*	0.011	
	Cd+XAV939 vs. Cd	-	ns	0.873	
	gap43	One-way ANOVA	***	F=61.99 P<0.001	
	CTRL vs. Cd	Dunnett's multiple comparisons	**	0.004	
	Cd+TWS119 vs. Cd	-	ns	0.135	
	Cd+XAV939 vs. Cd	-	**	0.003	
**Cell cycle distribution (Figure. 5B)**	**G1/G0**	One-way ANOVA	***	F=22.29 P<0.001	3
	Cd				
	CTRL vs. Cd	Dunnett's multiple comparisons	*	0.025	
	Cd+TWS119 vs. Cd	-	*	0.043	
	Cd+XAV939 vs. Cd	-	*	0.012	
	**S**	One-way ANOVA	**	F=13.14 P=0.002	
	Cd				
	CTRL vs. Cd	Dunnett's multiple comparisons	*	0.015	
	Cd+TWS119 vs. Cd	-	*	0.028	
	Cd+XAV939 vs. Cd	-	ns	0.329	
	**G2/M**	One-way ANOVA	***	F=30.26 P<0.001	
	Cd				

	CTRL vs. Cd	Dunnett's multiple comparisons	***	<0.001	
	Cd+TWS119 vs. Cd	-	**	0.002	
	Cd+XAV939 vs. Cd	-	ns	0.204	
**The number of AO positive cells (Figure. 5D)**		One-way ANOVA	***	F=199.9 P<0.001	10
	Cd				
	CTRL vs. Cd	Dunnett's multiple comparisons	***	<0.001	
	Cd+TWS119 vs. Cd	-	***	<0.001	
	Cd+XAV939 vs. Cd	-	**	0.001	
**The expression profiles of apoptotic related genes (Figure. 5E)**	***bcl***	One-way ANOVA	***	F=17.71 P<0.001	3
	Cd				
	CTRL vs. Cd	Dunnett's multiple comparisons	*	0.021	
	Cd+TWS119 vs. Cd	-	*	0.014	
	Cd+XAV939 vs. Cd	-	*	0.044	
	p53	One-way ANOVA	***	F=40.71 P<0.001	
	Cd				
	CTRL vs. Cd	Dunnett's multiple comparisons	**	0.003	
	Cd+TWS119 vs. Cd	-	**	0.004	
	Cd+XAV939 vs. Cd	-	***	<0.001	
	bax	One-way ANOVA	***	F=68.49 P<0.001	
	Cd				
	CTRL vs. Cd	Dunnett's multiple comparisons	***	<0.001	

	Cd+TWS119 vs. Cd	-	***	<0.001	
	Cd+XAV939 vs. Cd	-	ns	0.368	
	cas8	One-way ANOVA	***	F=89.78 P<0.001	
	Cd				
	CTRL vs. Cd	Dunnett's multiple comparisons	***	<0.001	
	Cd+TWS119 vs. Cd	-	***	<0.001	
	Cd+XAV939 vs. Cd	-	ns	0.216	
	cas3a	One-way ANOVA	***	F=27.86 P<0.001	
	Cd				
	CTRL vs. Cd	Dunnett's multiple comparisons	*	0.045	
	Cd+TWS119 vs. Cd	-	**	0.008	
	Cd+XAV939 vs. Cd	-	*	0.018	
**Microglia surface (Figure. 6B)**		One-way ANOVA	***	F=22.86 P<0.001	
	Cd				8
	CTRL vs. Cd	Dunnett's multiple comparisons	***	<0.001	8
	Cd+TWS119 vs. Cd	-	**	0.008	8
	Cd+XAV939 vs. Cd	-	*	0.034	7
**Microglia Sphericity (Figure. 6C)**		One-way ANOVA	***	F=8.484 P<0.001	
	Cd				8
	CTRL vs. Cd	Dunnett's multiple comparisons	***	<0.001	8
	Cd+TWS119 vs. Cd	-	**	0.003	8

	Cd+XAV939 vs. Cd	-	ns	0.85	7
**Microglia Volume (Figure. 6D)**		One-way ANOVA	***	F=23.20 P<0.001	
	Cd				8
	CTRL vs. Cd	Dunnett's multiple comparisons	***	<0.001	8
	Cd+TWS119 vs. Cd	-	*	0.021	8
	Cd+XAV939 vs. Cd	-	*	0.011	7
**Number of microglia in optic tectum (Figure. 6F)**		One-way ANOVA	***	F=12.43 P<0.001	
	Cd				11
	CTRL vs. Cd	Dunnett's multiple comparisons	***	<0.001	8
	Cd+TWS119 vs. Cd	-	**	0.001	8
	Cd+XAV939 vs. Cd	-	ns	0.997	8
**Microglia and inflammation related genes (Figure 6G)**	***irf8***	One-way ANOVA	***	F=24.16 P<0.001	3
	Cd				
	CTRL vs. Cd	Dunnett's multiple comparisons	**	0.002	
	Cd+TWS119 vs. Cd	-	**	0.006	
	Cd+XAV939 vs. Cd	-	ns	0.228	
	apoeb	One-way ANOVA	***	F=77.87 P<0.001	
	Cd				
	CTRL vs. Cd	Dunnett's multiple comparisons	***	<0.001	
	Cd+TWS119 vs. Cd	-	***	<0.001	
	Cd+XAV939 vs. Cd	-	ns	0.482	

	tnf-a	One-way ANOVA	***	F=33.02 P<0.001	
	Cd				
	CTRL vs. Cd	Dunnett’s multiple comparisons	***	<0.001	
	Cd+TWS119 vs. Cd	-	***	<0.001	
	Cd+XAV939 vs. Cd	-	ns	0.062	

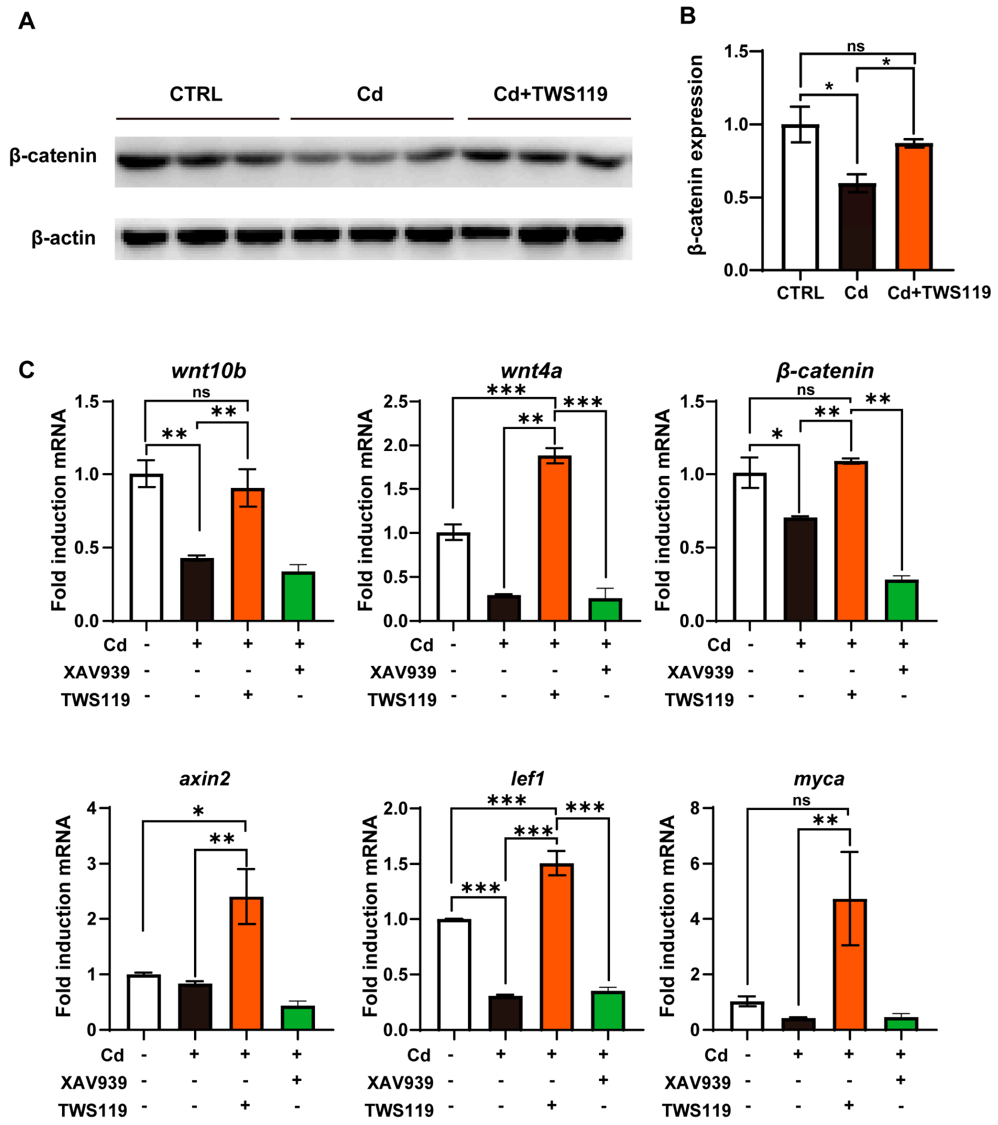

Figure S1. Effects of TWS119 and XAV939 on Wnt signaling pathway related proteins and genes.

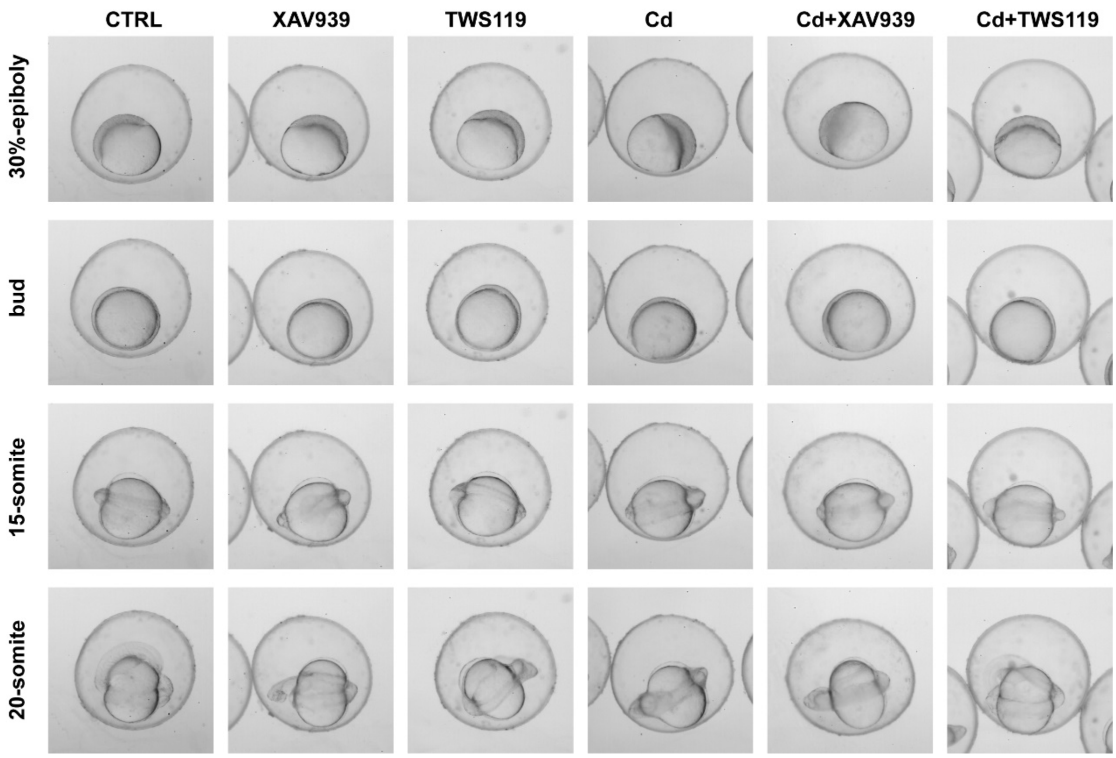

Figure S2. Representative images of high-throughput time-lapse tracking of zebrafish early development. See also **Video S1**.

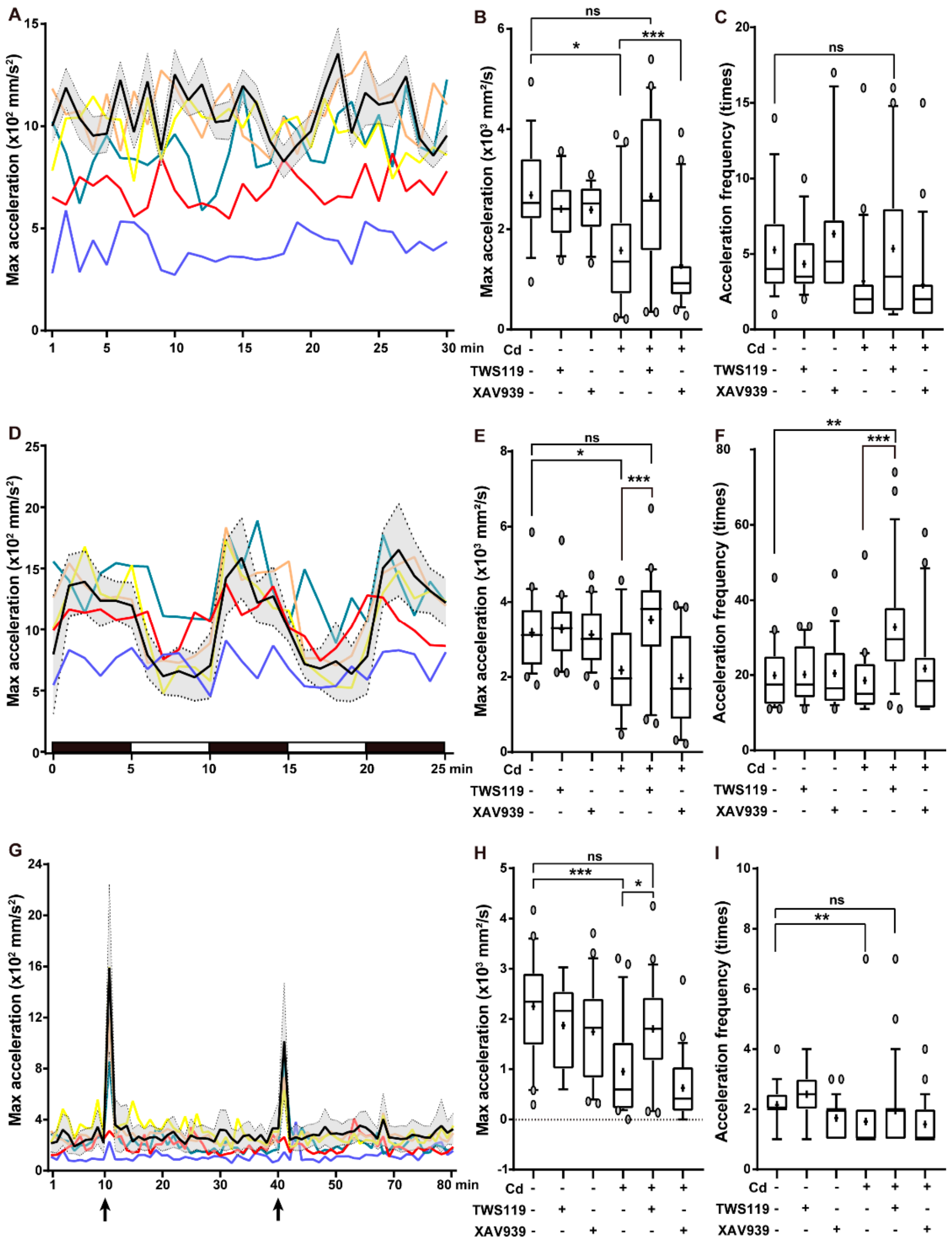

Figure S3. Zebrafish locomotor activities and reactivity to light-dark/vibration stimulation in different groups. Swimming distance, velocity of 6 dpf zebrafish larvae in 30 minutes **(A-C)**, subjected to light-dark cycles **(D-F)** and vibration stimulation in every 30 s (as indicated by the arrows below) **(G-I)**. Left panels were plotted in 5 min or 10 s time bin and right panels were plotted with average values of the whole-tracking. Each experiment was performed independently three times. The values are presented as median (line) and mean (+), while whiskers show 90% confidence levels in boxplots. Nonparametric Kruskal-Wallis test or one-way ANOVA followed by multiple comparisons test results are reported in **Table S2**. Significant differences are indicated by asterisks (***p < 0.001, **p < 0.01, *p < 0.05).

Video S1. High-throμghput time-lapse imaging to track the early development of zebrafish embryos.
